# On Scarlatina and Its Treatment

**Published:** 1859-04

**Authors:** E. Bishop

**Affiliations:** Devonport


### EXTRACTS.

### ON SCARLATINA AND ITS TREATMENT.

By E. Bishop, M.D., Devonport.

If we would have a science and the art founded thereon, to become more and more pure, we must direct our studies in the way which our present acquaintance with the science points out to us. If the way be one of theory, we must theorize; if it be one of practice and observation, we must observe and investigate. Medicine is essentially a science of facts. The store of facts is gradually accumulating, and has already, indeed, increased to such an extent as to render the teaching necessarily more and more practical, and to show that things, not words *en masse*, form the material with which the practitioner's mind must be supplied. The more medicine is made to partake of the real, the more prominent and bright will it shine.

The foregoing I saw in print some time since, and the remarks appear applicable to the following. It is desirable that contributions to the medical press, however trifling they may appear to some, should be published, especially respecting any new or successful treatment in serious diseases. It is the duty of the practitioner to record his method of treating disease. even if it does not appear strictly orthodox, or in accordance with the prescribed methods. If we had to rest entirely upon the authority of even our best authors, we should fall miserably short; for the numerous opinions given as to the nature and treatment of this and other diseases are so contradictory and conflicting as to mislead the matured as well as the juvenile practitioner. Or, to quote the language of Dr. Gilmour, "My young brethren in medicine must not trust implicitly to what they read in books; many of them are truly valuable and trustworthy, but others (and of these there is a large number) are written to suit a purpose, and contain trash."

I beg to state as briefly as possible the treatment I have adopted in this disease during the last nine months. There is nothing original in it, so far as it relates to myself. I know friends who have tried it with marked success. Scarlatina has been rife in this town for the last nine months, and has proved fatal to a large number of children belonging to all classes of society. In many families, one, two, and even three have succumbed to it. I applied to the Registrar for the exact number of deaths from this disease in the three quarters ending March, June, and September, 1858, and he has been kind enough to supply me with a return, by permission of the Registrar-General. In the first quarter of the present year, the deaths from scarlatina reached 23; in the second, 44; and in the third, 27, in children under ten years of age.

In fifty-one cases of scarlatina scattered over the town, in children varying from two to ten years of age, my plan of treatment has been tonics from the commencement (*i. e.* from my first visit), either the citrate of iron, or the tincture of the sesquichloride, in the usual full doses; and I have every reason to be satisfied with the result, having lost but one case. I made no difference in *the plan of treatment* even when serious complications presented. In many the fever was intense, the inflammation of the throat severe, and pain in swallowing very considerable. Four children in one house, in Cannon street, had scarlatina anginosa in an aggravated form, being attended with an acrid discharge from the ears and nostrils. In one case deafness has remained nearly permanent. I entirely discarded the application of strong caustics to the throat and tonsils, which many years' experience has taught me is injurious in very young children, and calculated to do more harm than good, to say nothing of the injury and difficulty attending the operation. External applications to the throat I found most beneficial—either the compound camphor liniment, oil and hartshorn, or turpentine sprinkled on a strip of flannel, previously wrung out of hot water, and applied several times during the twenty-four hours. Inhaling the steam of hot water gave much relief, as it generally does. The children I have been called upon to treat have long belonged to the poorer class; the diet necessarily simple; in severe cases, broths, beef-tea, milk, jelly, and wine were recommended, and procured, if possible. One gratifying result in the treatment of scarlatina with iron, as far as my experience carries me, is that the children, with few exceptions, escaped that serious and frequent sequel—anasarca.

I have seen two cases of diphtheria following scarlatina during the epidemic, in children four and six years of age. The first child had been convalescent a week or ten days, and I must confess, I could not understand the cause of the relapse. After a few days, I suspected diphtheria; the child would never allow me nor the parents to examine the state of the throat, although rough usage was resorted to more than once or twice. During the time it suffered extreme prostration, and was supported by wine and beef-tea; it also took a mixture containing the tincture of the sesquichloride of iron. This child ultimately coughed up the membrane characteristic of diphtheria. A fit of vomiting and coughing came on at a time when it appeared beyond hope; but when the membrane was released, the child was relieved, and gradually rallied.

A remarkable case under my care was that of a boy, aged four years, belonging to the Royal Naval and Military Free Schools. I first saw him twelve weeks ago from the date of this communication; he had then scarlatina anginosa, from which he recovered sufficiently to enjoy a walk. About a fortnight (to use his own words) "pimples coming out of his face and body." On visiting him, this proved to be variola discreta. He had been successfully vaccinated, judging from the cicatrices in his arm. The little fellow suffered severely; he had not regained his strength from the debilitating consequences of the previous illness. He was kept up by wine, beef-tea, ammonia and bark, as maturation of the pustules went on but slowly. He recovered from this attack, but not sufficiently to return to school, when I was requested to visit him, as he had hooping-cough, which was and is now epidemic in this town; and this being complicated with pneumonia, terminated his existence a few days ago. There was something remarkable in the fact of this child having three of the most serious and fatal diseases of childhood in the short period of three months. One little girl who was in the habit of going to the house of the deceased is now under treatment for variola.

I trust the tonic treatment of scarlatina with iron may have a trial elsewhere, and prove as efficacious and successful as it has been with me. I conceive it is far better to prove by facts than to judge and condemn without a trial, simply because the treatment does not harmonize with the doctrine laid down by our popular authors and preceptors.—*London Lancet.*

